# Apoptosis and failure of checkpoint kinase 1 activation in human induced pluripotent stem cells under replication stress

**DOI:** 10.1186/s13287-016-0279-2

**Published:** 2016-01-25

**Authors:** Joelle A. Desmarais, Christian Unger, Ivan Damjanov, Mark Meuth, Peter Andrews

**Affiliations:** Centre for Stem Cell Biology, Department of Biomedical Science, University of Sheffield, Western Bank, Sheffield, S10 2TN UK; Institute for Cancer Studies, Department of Oncology, School of Medicine, University of Sheffield, Beech Hill Road, Sheffield, S10 2RX UK; Department of Pathology, University of Kansas Hospital, 3901 Rainbow Blvd, Kansas City, KS 66160 USA

**Keywords:** Induced pluripotent stem cells, Integration-free reprogramming, Checkpoint kinase 1, Apoptosis, Replication stress, Ataxia telangiectasia and rad3-related, Ataxia telangiectasia mutated, Genomic instability

## Abstract

**Background:**

Human induced pluripotent stem (hiPS) cells have the ability to undergo self-renewal and differentiation similarly to human embryonic stem (hES) cells. We have recently shown that hES cells under replication stress fail to activate checkpoint kinase 1 (CHK1). They instead commit to apoptosis, which appears to be a primary defense mechanism against genomic instability. It is not known whether the failure of CHK1 activation and activation of apoptosis under replication stress is solely a feature of hES cells, or if it is a feature that can be extended to hiPS cells.

**Methods:**

Here we generated integration-free hiPS cell lines by mRNA transfection, and characterised the cell lines. To investigate the mechanism of S phase checkpoint activation, we have induced replication stress by adding excess thymidine to the cell culture medium, and performed DNA content analysis, apoptosis assays and immunoblottings.

**Results:**

We are showing that hiPS cells similarly to hES cells, fail to activate CHK1 when exposed to DNA replication inhibitors and commit to apoptosis instead. Our findings also suggest the Ataxia Telangiectasia Mutated pathway might be responding to DNA replication stress, resulting in apoptosis.

**Conclusion:**

Together, these data suggest that the apoptotic response was properly restored during reprogramming with mRNA, and that apoptosis is an important mechanism shared by hiPS and hES cells to maintain their genomic integrity when a replication stress occurs.

## Background

Human induced pluripotent stem (hiPS) cells have the ability to undergo self-renewal and differentiation similarly to human embryonic stem (hES) cells. However, instead of being derived from embryos, hiPS cells are produced through reprogramming from somatic cells of any individual and are therefore the ultimate source for personalized cells. hiPS cells were initially reprogrammed using retroviral vectors overexpressing a set of transcription factors found expressed at high levels in hES cells (i.e., octamer-binding transcription factor 3/ 4 (OCT4), sex determining region Y-box 2 (SOX2), kruppel-like factor 4 (KLF4), and C-MYC) [1]. However, retroviral vectors leave a footprint in the resulting induced pluripotent stem (iPS) cells that could cause insertional mutagenesis and oncogene activation, which makes them undesirable for clinical applications. For this reason, integration-free and virus-free methods have been developed to reduce this risk and open the way for clinical-grade iPS cell lines. In particular, mRNA-based reprogramming completely abolishes the risk of any DNA footprint and allows for efficient and fast reprogramming [2].

The ataxia telangiectasia and rad3-related (ATR) pathway is the primary response to replication stress in tumor models. The ATR complex assembles at stalled replication fork and activates checkpoint kinase 1 (CHK1). CHK1 then induces a DNA damage response cascade, resulting in cell cycle arrest, an inhibition of origin firing, and a stabilization of replication forks (reviewed in [3, 4]). Knockout or inhibition of CHK1 during DNA replication stress induces single-stranded DNA (ssDNA) stretches, DNA damage, and genomic instability [5, 6]. Recently, we have shown that hES cells under replication stress fail to activate CHK1. They instead commit to apoptosis without forming ssDNA, which appears to be a primary defense mechanism against genomic instability [7].

It is not known whether the failure of CHK1 activation and activation of apoptosis under replication stress is solely a feature of hES cells, or whether it is a feature that can be extended to hiPS cells. Given the vast interest for the potential use of hiPS cells in clinical therapies, it is important to know whether hiPS cells are able to protect themselves against genomic instability in a similar way to hES cells, knowing that hiPS cells can differ from hES cells in their epigenetic profiles and in functionalities [8, 9]. Here we generated integration-free hiPS cell lines by mRNA transfection to investigate the mechanism of S-phase checkpoint activation within those cells.

## Materials and methods

### Cell lines and treatments

This study employed the Shef5 hES cell line, derived at the University of Sheffield [10], the CRL-2429 human fetal foreskin fibroblasts (HFF) and the HCT116 colon cancer cell line (ATCC, Manassas, VA, USA). Thymidine (TdR) (2 mM; Sigma, St. Louis, MO, USA) was used to induce replication stress.

### Reprogramming and iPS maintenance

HFF were grown in FBSm media consisting of Dulbecco’s modified Eagle’s medium (DMEM) + 10 % fetal bovine serum (FBS) and reprogrammed in a norm-oxygen environment using a Stemgent mRNA Reprogramming kit (Stemgent, Cambridge, MA, USA) following the manufacturer’s guidelines. Briefly, HFF were seeded onto inactivated human feeders cells in Pluriton™ medium (Stemgent), followed by daily transfections with modified mRNA encoding the five transcription factors OCT4, SOX2, KLF4, CMYC, and LIN28 while suppressing the intracellular immune response by adding interferon-binding protein B18R. Successfully reprogrammed colonies were selected by morphology and picked mechanically for the first three passages, and then propagated using Collagenase IV. hiPS cells were maintained on inactivated mouse embryonic fibroblast feeder cells in KSR medium (DMEM–F12 supplemented with 20 % KnockOut™ Serum Replacement (Life Technologies, Carlsbad, CA, USA ), 1 % non-essential amino acids, 1 % l-glutamine, 0.1 mM beta-mercaptoethanol, 4 ng/ml basic fibroblast growth factor).

The resulting hiPS cell lines were named mRNA-induced foreskin fibroblast (MIFF). The MIFF lines used for checkpoint analysis were between passages 10 and 20 and karyotypically normal (data not shown). MIFF1 and MIFF3 were registered online (http://hpscreg.eu/).

### Immunocytochemistry

Primary and secondary antibodies were diluted in phosphate-buffered saline + 10 % FBS. Antibodies used were anti-OCT3/4 (C-10, 1:100; SantaCruz, Santa Cruz, CA, USA, and concentrated supernatant from hybridomas anti-stage-specific embryonic antigen (SSEA)-1 (MC480-1, 1:10 [11]), anti-SSEA-4 (MC813-70, 1:10 [12]), and anti-TRA-1-81 (1:10 [13]). Images were taken on the InCell Analyzer platform (GE Healthcare, Little Chalfont, Buckinghamshire,U.K.).

### Reverse transcription-polymerase chain reaction (RT-PCR)

Total RNA was extracted using Trizol reagent (Life Technologies) following the manufacturer’s guidelines. A high-capacity cDNA reverse transcription kit (Applied Biosystems, Carlsbad, CA, USA) was used to generate cDNA. Subsequent RT-PCR reactions were carried out using Taq DNA polymerase (Life Technologies). Primers were as follows: beta-actin, forward TGAAGTGTGACGTGGACATC and reverse GGAGGAGCAATGATCTTGAT; alpha-fetoprotein (AFP), forward CGCTGCAAACGATGAAGCAAG and reverse AATCTGCAATGACAGCCTCAAG; brachyury, forward CGCATGATCACCAGCCACTG and reverse TTTAAGAGCTGTGATCTCCTCG; and paired box 6 (PAX6), forward AATAACCTGCCTATGCAACCC and reverse AACTTGAACTGGAACTGACACAC).

### Teratoma formation assay

Single iPS cells (5 × 10^6^–8 × 10^6^ cells) detached with Accutase (Life Technologies) were mixed with Matrigel and 3 × 10^5^ inactivated MEFs in a total volume of 100 μl. The cell mix was injected subcutaneously and mice were sacrificed after 9–12 weeks. Teratomas were dissected, fixed in formaldehyde, embedded, sectioned, and stained with hematoxylin and eosin (H & E).

### DNA content and apoptosis assay

DNA content was analyzed as described previously [14]. Acquisition of flow cytometric data was carried out with a CyAn ADP analyzer (Beckman Coulter, High Wycombe, UK), and FlowJo 7.6 software was used for analysis (TreeStar Inc., Ashland, OR, USA). AnnexinV staining was performed according to the manufacturer’s instructions (BD Bioscience, Franklin Lakes, NJ, USA).

### Immunoblottings

Antibodies and protocols were published previously [7]. P53S15 was obtained from Cell Signaling (Danvers, MA, USA, no. 9284 s, 1:1000;).

### Statistical analysis

Statistical analysis was conducted using JMP (SAS Institute, Cary, NJ, USA). A one-way analysis of variance was used to evaluate the variance between groups, and a Student’s *t* test was used to evaluate the statistical differences between control and experimental groups. *p <*0.05 was considered significant.

## Results

**Fig. 1:**
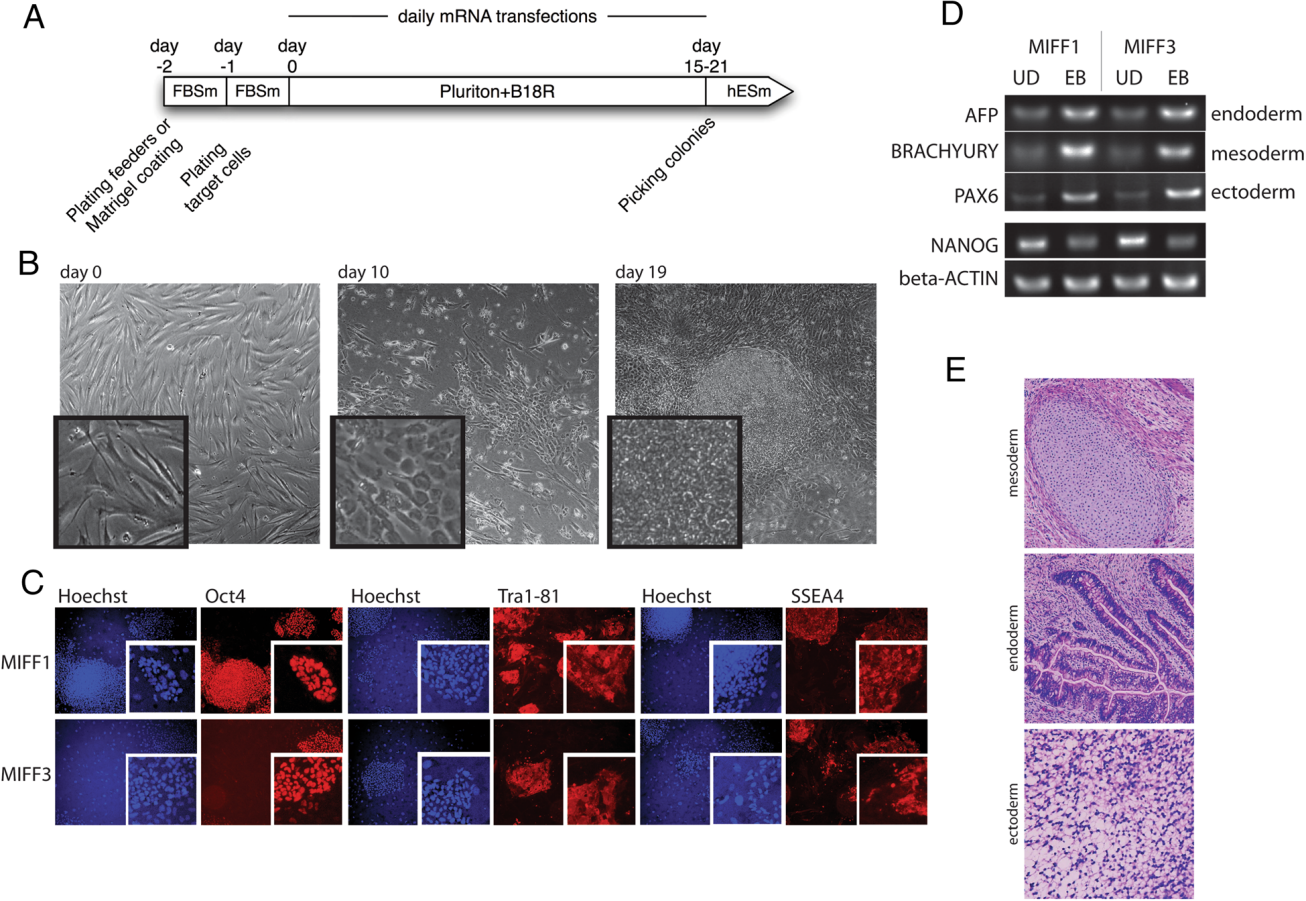
hiPS cells generated with an mRNA-based integration-free method display typical characteristics of hES cells. **a** Timeline and essential steps for the reprogramming of human fibroblasts into mRNA-induced iPS cells. Human fibroblasts were plated 1 day before the first transfection in FBSm media, on a human feeder-coated dish. Cells were transfected daily with synthetic mRNA encoding the factors OCT4, SOX2, KLF4, C-MYC, and LIN28 in Pluriton™ reprogramming medium plus B18R (all provided by Stemgent) and kept in norm-oxygen (21 %) conditions. iPS colonies appeared between days 15 and 21, when they were mechanically picked and moved onto feeders in KSR medium. **b** Morphological changes of human fibroblasts throughout the reprogramming period in norm-oxygen. Typical fibroblast morphology at the start (day 0), transitioning cells with epithelial morphology half-way (day 10), until embryonic stem cell-like colonies have formed (day 19). Magnification: 40× (*background images*), digital zoom (*smaller windows*). **c** hiPS cell lines express typical intracellular and extracellular pluripotency markers. Immunofluorescence staining with monoclonal antibodies of stem cell markers OCT4, TRA-1-81, and SSEA4 (*red*) and Hoechst DNA counterstain (*blue*) shown for iPS cell lines MIFF1 and MIFF3. Magnification: 40× (*background images*), digital zoom (*smaller windows*). **d** Established hiPS cells are able to differentiate and induce markers of three germ layers in a 7-day EB differentiation assay. Early ectoderm marker PAX6, mesoderm marker brachyury, and endoderm marker AFP are upregulated in day 7 EBs, as analyzed by RT-PCR. Nanog, a marker of undifferentiated hES cells, decreases its expression upon differentiation. Beta-actin is used as a housekeeping gene. **e** Representative images of H & E-stained microsections of a teratoma generated after injection of hiPS cells into immunocompromised mice. Teratomas were extracted 9–12 weeks after injection and fixed in formaldehyde before embedding and sectioning. Sections show the presence of cartilage (mesoderm), intestinal glandular-like structure (endoderm), and neural tissue (ectoderm), representing derivative of the three germ layers. Magnification: 160×. *AFP* alpha-fetoprotein, *UD* undifferentiated cells, *EB* embryoid body, *FBS* fetal bovine serum, *hES* human embryonic stem, *MIFF* mRNA-induced foreskin fibroblast, *PAX6* paired box 6, *SSEA* stage-specific embryonic antigen

We produced integration-free iPS cell lines from HFFs as summarized in Fig. 1a, using mRNA encoding five reprogramming factors, namely OCT4, SOX2, KLF4, C-MYC, and LIN28, and nuclear green fluorescent protein (GFP) as a transfection reporter [2]. Messenger RNA was transfected daily into fibroblasts until iPS cell colonies appeared, between days 15 and 21 (Fig. 1b). hiPS cell lines were characterized for their expression of stem cell markers and their ability to differentiate into derivatives of the three germ layers. The undifferentiated MIFF iPS cell lines expressed characteristic markers of undifferentiated pluripotent stem cells, OCT4, TRA-1-81, and SSEA4 (Fig. 1c) but not the differentiation marker SSEA1 (data not shown). When put through an embryoid body (EB) differentiation protocol, they upregulated the expression of differentiation markers, AFP (endoderm), brachyury (mesoderm), and PAX6 (ectoderm), indicating their ability to generate derivatives of the three germ layers (Fig. 1d). Further, in severe combined immunodeficiency (SCID) mice, the MIFF lines also formed teratomas that showed the presence of cartilage (mesoderm), intestinal glandular-like structure (endoderm), and neural tissue (ectoderm) (Fig. 1e). Additionally, we confirmed that MIFF iPS cell lines were karyotypically normal (46XY) and DNA fingerprinting established their parental origin from the HFF line (data not shown).

**Fig. 2:**
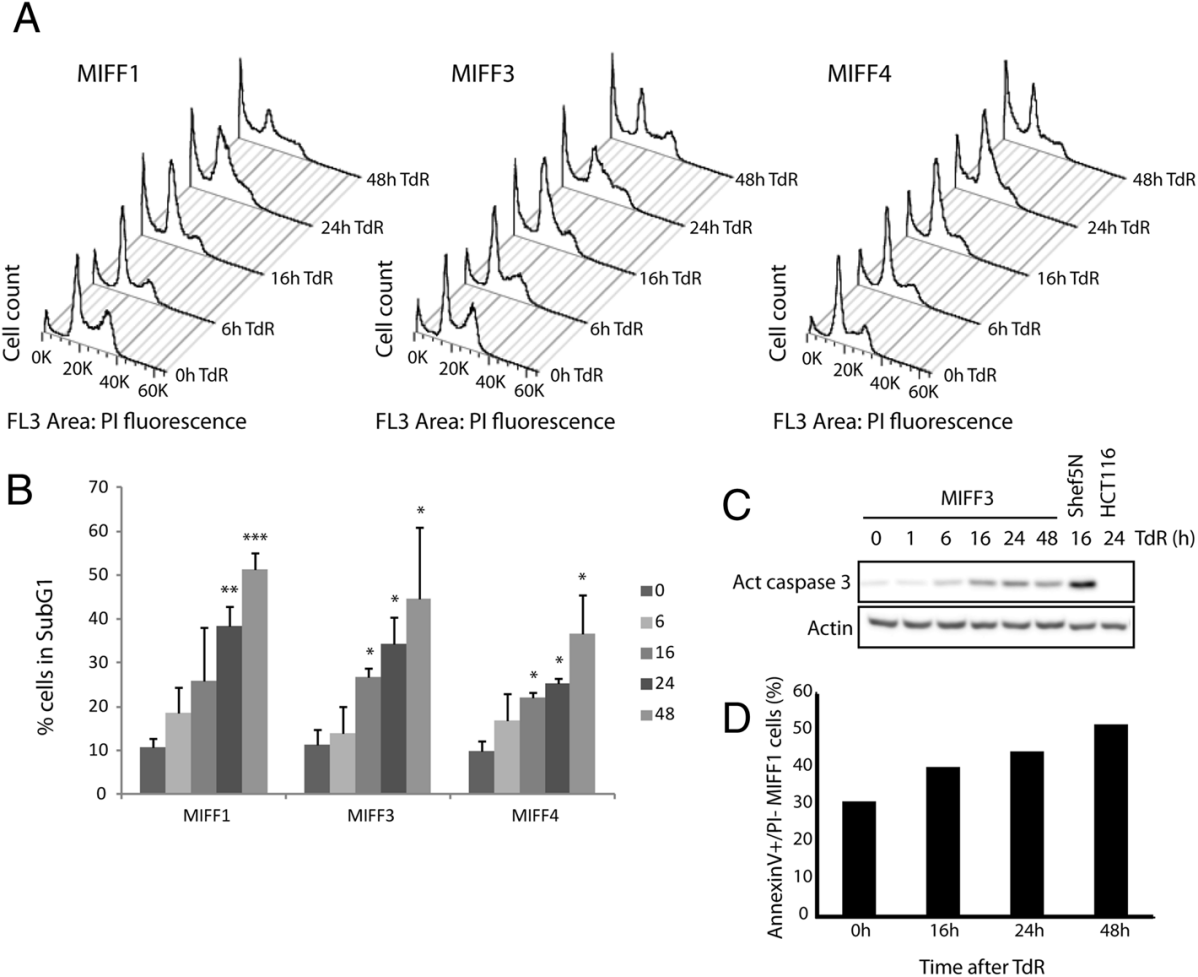
hiPS cells undergo apoptosis and no cell cycle arrest in response to replication inhibitor. **a** hiPS cell lines MIFF1, MIFF3, and MIFF4 show an increase in the sub-G1 fraction after TdR treatment as reflected by stacked PI profiles obtained by flow cytometry at different time points. hiPS cells show an early accumulation in the S phase but fail to reach G2 phase. **b** Graph depicting the increasing levels of the sub-G1 fraction determined from their PI profile, according to the time of TdR treatment, for each cell line MIFF1, MIFF3, and MIFF4. **c** Western blots showing an increased activation of caspase 3 protein level following TdR treatment. Beta-actin is presented as the control. Shef5N, a normal hES cell line, also show this increase in caspase 3 activation, while the somatic cell line HCT116 does not, in response to TdR. **d** Increased proportions annexinV^+^/PI^–^ MIFF1 cells, a marker of apoptosis, after TdR treatment. *p <0.05, **p <0.001, ***p <0.0001. *MIFF* mRNA-induced foreskin fibroblast, *PI* propidium iodide, *TdR* thymidine

The apoptotic response following DNA replication stress was investigated in three iPS cell lines (MIFF1, MIFF3, and MIFF4). Activation of the S-phase checkpoint was induced by adding excess TdR to the culture environment. The propidium iodide (PI) profile of MIFF3 and MIFF4 showed a significant increase in the sub-G1 population after 16 hours of TdR, and all three cell lines showed a significant increase after 24 hours of TdR treatment (Table 1, Fig. 2a, b). Concomitant with this increase in the sub-G1 population, the number of cells in the G1, S, and G2 phases were reduced in all three iPS cell lines (Table 1, Fig. 2a). In MIFF3 cells, an increase in active caspase 3 expression and an increment in annexinV^+^/PI^−^ cells in MIFF1 cells were both indicative of apoptotic cells (Fig. 2c, d). Similarly, Shef5N hES cells showed an increase in active caspase 3 expression after TdR treatment. These data suggest that iPS cells, like hES cells but unlike somatic tumor cells, undergo apoptosis after replication stress but do not sustain a cell cycle arrest.

**Table 1:** Cell cycle distribution of iPS cells treated with thymidine

	Thymidine				
	0 hours	6 hours	16 hours	24 hours	48 hours
MIFF1					
Sub-G1 phase	10.7 ± 2.2	18.5 ± 6.1	25.8 ± 12.5	38.5 ± 4.2**	51.1 ± 3.9***
G1 phase	33.2 ± 3.4	44.5 ± 0.6*	38.3 ± 4.2	24.5 ± 5.1	20.3 ± 1.5*
S phase	28.9 ± 1.4	20.2 ± 2.3*	23.7 ± 1.8	29.1 ± 4.8	18.7 ± 4.3*
G2 phase	25.6 ± 2.2	15.7 ± 4.4*	10.6 ± 8.1*	7.4 ± 2.9**	8.7 ± 1.6**
Total	98.3 ± 1.4	99.0 ± 1.5	98.4 ± 0.7	99.5 ± 1.4	98.9 ± 0.6
MIFF3					
Sub-G1 phase	11.4 ± 3.5	14.0 ± 5.9	26.7 ± 2.2*	34.4 ± 6.2*	44.5 ± 16.6*
G1 phase	30.3 ± 2.3	41.4 ± 3.8*	31.0 ± 4.3	22.4 ± 1.7	21.0 ± 5.0*
S phase	29.2 ± 0.8	29.1 ± 3.7	30.9 ± 3.1	30.9 ± 2.8	22.5 ± 4.8
G2 phase	27.3 ± 5.0	14.7 ± 0.3*	10.7 ± 1.7*	11.2 ± 4.5*	11.0 ± 8.8*
Total	98.2 ± 0.2	99.2 ± 1.4	99.2 ± 0.6	98.9 ± 0.5	98.9 ± 0.2
MIFF4					
Sub-G1 phase	9.7 ± 2.4	16.9 ± 5.9	22.1 ± 1.1*	25.2 ± 1.2*	36.8 ± 8.6*
G1 phase	40.2 ± 8.1	40.1 ± 4.2	38.4 ± 4.5	31.3 ± 6.9	27.0 ± 3.6
S phase	25.7 ± 2.5	26.1 ± 5.6	28.6 ± 2.6	31.4 ± 2.4	22.9 ± 5.7
G2 phase	22.3 ± 7.6	15.0 ± 4.0	9.1 ± 1.2	10.5 ± 5.8	11.9 ± 6.3
Total	97.8 ± 0.4	98.0 ± 0.5	98.1 ± 0.4	98.3 ± 0.1	98.5 ± 0.2

*p <0.05, **p <0.001, ***p <0.0001

*iPS* induced pluripotent stem, *MIFF* mRNA-induced foreskin fibroblast

**Fig. 3:**
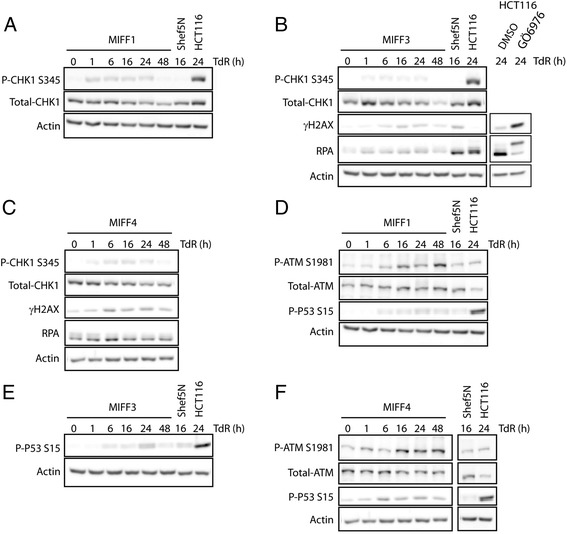
Activation of DNA damage response pathways in iPS cell lines in response to TdR. **a** MIFF1, **b** MIFF3, and **c** MIFF4 iPS cell lines show a reduced CHK1 activation in western blots, comparable with what is observed in the Shef5N normal hES cell line (**b**) and in contrast to the strong activation observed in HCT116 cells (**b**). In all iPS cell lines, there is a reduced γH2AX phosphorylation compared with that observed in HCT116 treated with the CHK1 inhibitor Gö6976 (**b**), indicating that DNA damage is not enhanced in these cell lines in response to replication inhibitor TdR, despite the absence of a clear CHK1 activation. In addition, RPA is not hyperphosphorylated in any iPS cell lines, suggesting that ssDNA formation is suppressed. In contrast, HCT116 cells treated with the CHK1 inhibitor Gö6976 (**b**) show a marked hyperphosphorylation of RPA. **d** MIFF1 and **f** MIFF4 activate ATM by phosphorylation of Ser1981 after TdR treatment. This is accompanied by the phosphorylation of P53 at S15 in MIFF1 (**d**), MIFF3 (**e**), and MIFF4 (**f**). *CHK1* checkpoint kinase 1, *DMSO* dimethyl sulfoxide, *γH2AX* γ histone 2AX, *MIFF* mRNA-induced foreskin fibroblast, *RPA* replication protein A, *TdR* thymidine

Next, we analyzed the activation status of the proteins CHK1, γ histone 2AX (γH2AX), and replication protein A (RPA), known to be signaling through the ATR pathway and S-phase checkpoint [3]. All three iPS cell lines displayed reduced levels of pSer345-CHK1 following TdR, compared with the levels observed in the HCT116 control cell line (Fig. 3a–c). The low levels of pSer345-CHK1 were comparable with those observed in Shef5N (Fig. 3a, b). Despite the absence of CHK1 activation, the total CHK1 protein was expressed at constant levels after TdR treatment.

RPA binds ssDNA and is hyperphosphorylated following DNA damage or genetic stress [15], and γH2AX is phosphorylated at the sites of stalled replication fork [16]. In U-2-OS osteosarcoma cells, inhibition of CHK1 results in the phosphorylation of RPA and γH2AX [6]. Therefore, we asked whether the absence of CHK1 signaling caused a similar increase in RPA and γH2AX activation in hiPS cells. We found that TdR did not significantly induce the hyperphosphorylation of RPA and nor did it induce the activation of γH2AX in hiPS cells (Fig. 3b, c), while both were induced in control HCT116 cells in the presence of a CHK1 inhibitor, Gö6976 (Fig. 3b).

In some situations, ATR response can be substituted by the ataxia telangiectasia mutated (ATM) response (reviewed in [17]). We therefore examined the ATM pathway in iPS cells, and indeed found a delayed activation in MIFF1 and MIFF4 cell lines following TdR treatment (Fig. 3d, f). This was accompanied by a mild activation of P53 phosphorylation at Ser 15 (Fig. 3d–f). This activation could suggest a possible role of the ATM pathway in replication stress-induced apoptosis.

## Discussion

To our knowledge, this is the first study examining the S-phase checkpoint response in hiPS cells, demonstrating that while there is a suppression of CHK1 activation, there is also an increase in apoptosis in response to DNA replication stress. These results are similar to what we have described previously in hES cell lines [7].

While CHK1 is expressed, it is only weakly activated in response to TdR in iPS cells, when compared with the CHK1 response observed in HCT116 cells. Different levels of suppression are observed amongst different iPS cell lines, suggesting that CHK1 phosphorylation is suppressed, but not absent. A similar observation has been made in hES cells [7]. Moreover, CHK1 can be activated in hES cells following different types of damage (e.g. irradiation induced) [7, 18], suggesting that the lack of response to replication stress could be the result of a failure to form the ssDNA that triggers CHK1 activation in such cells. Our findings also suggest the ATM pathway might alternatively be responding to DNA replication stress, resulting in apoptosis.

Most studies reporting CHK1 activation in response to replication stress in somatic cells have been conducted in tumor models. Recent studies conducted on normal cells, notably an immortalized human diploid fibroblast cell line and an immortalized urothelial cell line, suggest that the ATR–CHK1 pathway response is delayed or simply not activated in response to replication stress [19, 20]. However, in contrast to what is observed in hES and hiPS cells, these nontumor cell lines rapidly activate ATM, which results in a cell cycle arrest in the G1 phase, while apoptosis is not induced [4]. The delay or absence of ATR–CHK1 response in fibroblast and urothelial cells, and the similar response in hES/hiPS cells, might suggest that these cell types process arrested replication forks using a different mechanism from that used by tumor cells [4]. More importantly, these data suggest that somatic cells do not share the same apoptotic mechanism as hES and iPS cells in response to replication stress, and the apoptotic response was properly restored during reprogramming with mRNA.

## Conclusion

Genomic integrity is of primary importance when considering the use of iPS cells in cellular therapies. While hiPS can be different on the epigenetic level, or can differ in their capacity for differentiation, this report suggests that apoptosis is an important mechanism shared by hiPS and hES cells to maintain their genomic integrity when a replication stress occurs.

## Abbreviations

AFP: Alpha-fetoprotein
ATM: Ataxia telangiectasia mutated
ATR: Ataxia telangiectasia and rad3-related
CHK1: Checkpoint kinase 1
DMEM: Dulbecco’s modified Eagle’s medium
EB: Embryoid body
FBS: Fetal bovine serum
GFP: Green fluorescent protein
γH2AX: γ Histone 2AX
H & E: Hematoxylin and eosin
hES: Human embryonic stem
HFF: Human fetal foreskin fibroblasts
hiPS: Human induced pluripotent stem
iPS: Induced pluripotent stem
KLF4: Kruppel-like factor 4
MIFF: mRNA-induced foreskin fibroblast
OCT4: Octamer-binding transcription factor 3/ 4
PAX6: Paired box 6
PI: Propidium iodide
RPA: Replication protein A
SCID: Severe combined immunodeficiency
SOX2: Sex determining region Y-box 2
ssDNA: Single-stranded DNA
SSEA: Stage-specific embryonic antigen
TdR: Thymidine
UD: Undifferentiated cells
